# Efficacy of a pheromone-impregnated collar in controlling feline problem behaviors, and an assessment of adverse events associated with collar use

**DOI:** 10.3389/fvets.2024.1468634

**Published:** 2024-12-19

**Authors:** Sarah Endersby, Charlotte Billy, Xavier De Jaeger

**Affiliations:** Ceva Santé Animale, Libourne, France

**Keywords:** pheromone, cat, problem behavior, collar, FELIWAY^®^

## Abstract

The aim of this study is to assess a pheromone complex-impregnated collar in the control of feline problem behaviors, and to assess the ease of use and tolerance of the collar compared with a regular collar. Six hundred and twenty-four cats from 459 households with one or more of four problem behaviors (problem urination, scratching, fear, or inter-cat conflict) were recruited to a 28-day study. Households were randomly assigned so that each cat received either a pheromone-impregnated polymer collar (containing 13% FELIWAY^®^ Optimum) or a control regular silicone collar. Caregivers completed online questionnaires at recruitment and on days 7, 14, and 28 documenting the frequency and intensity of the problem behavior in the previous 7 days, and documenting any loss, problems and tolerance of the collar (pheromone-impregnated or control). Complete data was available for 491 cats for assessment of efficacy. Compared with the control collar, the pheromone collar produced significantly better improvement in problem urination (*P* = 0.0172), scratching (*P* = 0.0013), and inter-cat conflict (*P* = 0.0029). There was also a greater, but non-significant improvement in problem fear scores (*P* = 0.063). Collars had been removed definitively or lost from 12.1% of cats, for various reasons, by the end of the study, and potential adverse reactions were reported in 27.2% of cats, but again, with no difference in the overall frequency reported between the two collar groups. In a controlled study, a FELIWAY^®^ Optimum-impregnated collar was shown to be effective in helping to manage a range of problem feline behaviors. The use of the pheromone collar was not associated with a higher level of adverse reaction reporting, but caregiver removal or loss of collars may present an obstacle for effective therapy through this means.

## Introduction

Feline problem behaviors are a frequent caregiver complaint among cats presented to veterinary clinics and commonly include anxiety or fear, aggression, urination problem, furniture scratching, vocalization, compulsive behaviors, pica, over activity, and predation (1–7). Many undesirable behaviors are part of the normal feline repertoire, they can be problematic for caregivers and can be indicators of an underlying stressful environment (4). The important and problematic nature of these behaviors is highlighted by them being reported as a very common reason for cats to be relinquished to rehoming organizations and for the failure of successful rehoming (8).

Several patented synthetic pheromones have been developed for use in cats including a feline facial pheromone, a feline appeasing pheromone and a feline inter-digital pheromone (respectively FELIWAY^®^ *Classic*, FELIWAY^®^ *Friends*, and Feliscratch^®^, Ceva Santé Animale), and these have been used successfully to help manage a variety of problem behaviors in cats (9, 12, 14–17, 29). Recently, the same group that developed these synthetic pheromones used an *in silico* binding modeling of the feline VR1 pheromone receptor in the vomeronasal organ to develop a new patented complex of synthetic pheromone molecules designed to address a broader range of behavioral problem with a single product (19, 20), and this is now available as a proprietary plug-in diffuser (FELIWAY^®^ *Optimum*, Ceva Santé Animale). In a previous uncontrolled open label study of the commercial diffuser (18), significant improvements were reported in problem urination, scratching, fearful behavior and inter-cat aggression, but the current report is the first placebo-controlled study to evaluate the new pheromone complex on those behaviors.

Managing behavioral problems is complex, often requiring an integrated approach with the use of environmental change, behavioral modification and sometimes the use of pharmacological agents (4). Since the introduction of the first commercially available synthetic feline pheromone in 1996 (FELIWAY^®^ *Classic*, Ceva Santé Animale), pheromones have also been successfully used as part of problem behavior management (9–17). Recently, a new proprietary complex of synthetic feline pheromone molecules at a specific ratio has been introduced (FELIWAY^®^ *Optimum*, Ceva Santé Animale) designed to address a broader range of behavioral modifications with a single product (18–20). An initial open-label uncontrolled study was published evaluating the efficacy of this new complex as a plug-in diffuser (17), with encouraging results. In dogs, pheromone-impregnated collars have been successfully used both in the control of insects (21), and also canine problem behaviors (22–24), but to date there are no published studies employing this approach in cats. The purpose of the current study was 2-fold. Firstly, to evaluate the new pheromone complex in a randomized, placebo-controlled study using a prototype therapeutic collar to deliver the complex; and secondly, to assess the adverse events (AE) reported with the use of the prototype collar in comparison with a control (regular) collar, as limited data currently exists on the tolerance of collars by cats (25).

Our predictions were that (1) FELIWAY^®^ *Optimum*-impregnated collars would be superior to control (regular) collars in helping to improve feline problem behaviors; and that (2) differences in adverse events might be seen between the two collars because of their differing physical properties.

## Materials and methods

### Study population

Cat caregivers were recruited from IMASENS proprietary databases of caregivers in France. IMASENS is a company that performs online surveys. Cat caregivers on the IMASENS database that resided in France were emailed an advertisement to participate in a research study. The online recruitment questionnaire was accessed via a link. Participation was linked to their identity, and respondents provided consent before being able to participate in the questionnaire. Caregivers had to ≥18 years of age and to be the main carer for the cat(s). Households had to meet the following inclusion criteria for the study:

- One or two cats had to be present, with each cat being >6 months of age.
- Each cat had to weigh ≥2.5 kg and had worn and tolerated a collar previously.
- There had to be ≥1 scratching post for the cat(s); and ≥1 litter tray and ≥1 food bowl per cat.
- At least one cat had to be displaying at least one of the four following problem behaviors, with minimal frequency of at least 2 per week and must have persisted for a ≥1 month:
  - Problem urination (defined as urinating away from the litter box, indoors, in a standing position and against a vertical surface).
  - Problem scratching (defined as scratching vertical surfaces indoors, other than scratching posts, such as furniture and curtains).
  - Problem fear (defined as fearful behavior such as hiding in response to visitors, unusual or loud noises, unusual situations etc.).
  - Problem inter-cat conflict (defined as signs of conflict, fighting, intimidation between cats when indoors and when there were two cats in the household).

Those four behaviors and definitions have been chosen based on previous publication (18, 26).

Caregivers and cats were excluded from the study if:

- The cat(s) had any concomitant veterinary-diagnosed disease or health problem.
- Any pheromones or calming products (including nutraceuticals) had been used within the preceding 6 months.
- Any cat had been housed away from the home within the previous 15 days.
- The caregiver anticipated being away from home for more than 2 days per week during the study.

All study participants provided full informed consent meaning that they were aware of the possibility to receive a placebo and of possible adverse events by clicking on the acceptance button after receiving all of the information. Caregivers were asked not to change their normal interactions with their cat(s), and to avoid changing the environment as far as possible during the study. The data collection period was between April and May 2021. At the end of the study, caregivers received incentives for their participation.

### Study design

**Figure 1:**
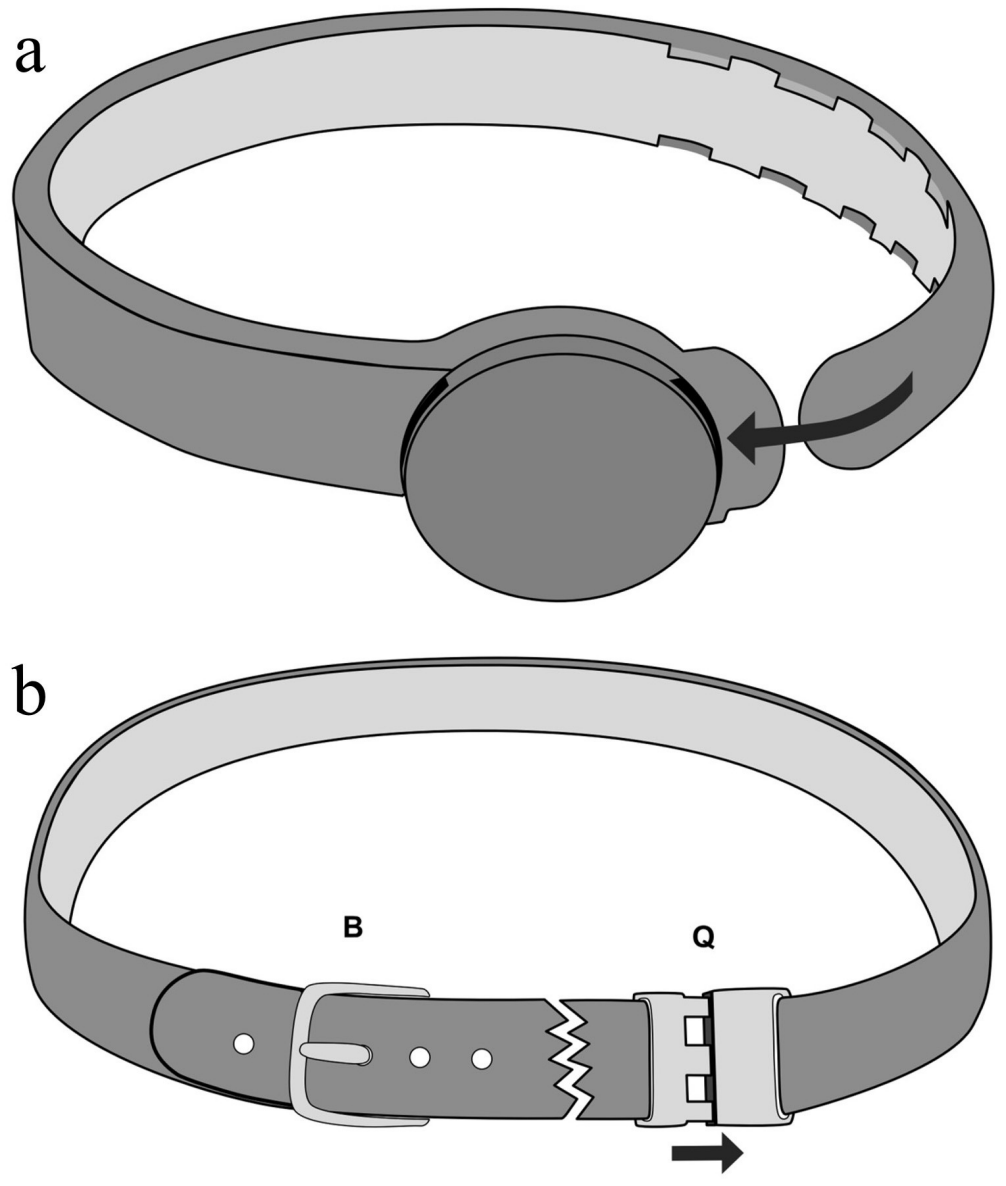
Diagrammatic demonstrating the fastening mechanisms in the test and control collars. **(A)** Diagrammatic representation of the pheromone-impregnated pheromone collar (PC) illustrating the molded notches/ratchet mechanism to hold the collar in place. **(B)** Diagrammatic representation of the silicone control collar illustrating the buckle (B) adjustment and the quick-opening fastening (Q).

The study was a blinded, randomized, placebo-control design. Households were randomly assigned to one of two groups—the “Pheromone Collar” (PC) or the “Control Collar” (CC) group using a randomization list, so that if there were two cats in the house, both received the same collar. The randomization was performed by blocks of 6 (equal collar distribution each 6 households) with SAS v9.4 (SAS Institute). The collars were distributed by ascending order according to the principal behavior sign (1–120 for indoor scratching, 121–240 for indoor urine marking, 241–360 for excessive fear, and 361–480 for difficulty of cats' cohabitation). To maintain the blinding condition, caregivers received only one kind of collar with no possibilities of comparison with the other. The PC was a prototype plasticized polymer collar impregnated with 13% FELIWAY^®^ *Optimum*, designed to provide a slow release of the product over 28 days. Because incorporation of the pheromone complex made the collar softer and more pliable, a CC of the same material without the synthetic pheromone mix was unsuitable. Instead, a commercially available silicone collar was chosen that had a similar flexibility. The width and thickness of the collars were 10 x 3 mm for the PC, and 12 x 1 mm for the CC. The PC had a molded loop through which the free end of the collar was passed that incorporated a molded notch/ratchet system (corresponding to notches in the free end of the collar) to adjust and maintain the collar length when fitted to the cat (Figure 1A), whereas the CC incorporated a plastic pin-buckle to adjust the collar length (Figure 1B). Both collars incorporated a safety breakaway mechanism (a weak point was incorporated into the PC polymer, and a plastic clip was incorporated into the CC which allowed both quick removal of the collar (without adjusting the buckle) and acted as a break point (Figure 1B).

Collars were delivered directly to caregivers with instructions on how to place them safely, with adjustment so that two fingers (but no more) could be passed between the collar and the cat's skin (24). For the PC only, caregivers were also instructed to cut off any excess length of collar (at the free end) after it had been secured in place. Caregivers were asked to check (and adjust if necessary) the collar fit every time they completed a questionnaire. If the cat was already wearing a collar at the start of the study, caregivers could choose whether to replace it, or use the study collar alongside the existing collar.

Study data were collected through online questionnaires. Inclusion and exclusion criteria were checked using a screening questionnaire, with an aim to recruit ≥240 cats to each treatment group (PC and CC), distributed evenly within the four problem behavior groups. This was based on power calculations indicating this number would be sufficient to detect a significant difference in outcomes (problem behavior index) with an 80% power and a two-sided alpha level of 5% (given an anticipated average reduction in scores of 60 and 25% and a variance of 45% in the PC and CC groups, respectively).

During the study (days 0, 7, 14, and 28), online questionnaires were used to collect data on the cat's recent behavior, and on the placement and tolerance of the collar along with any problems or defects noted with the collar (see Supplementary material). On each occasion caregivers completed questionnaires “blind,” without access to their previous answers.

### Efficacy of the collar

Data were collected about the problem behavior(s) during the preceding 7 days on each occasion a questionnaire was completed (see Supplementary material) which were similar to a recent study (26) using the scale named CABIAS^TM^ (Cat Behavior Issues Assessment Scale). The data of this study were collected prior to the validation of CABIAS, so in consequence some ameliorations which were added during the development of the scale are not included in this study. The data collected:

- The frequency of the problem behavior on a 7-point numerical scale.
- The perceived intensity or severity of the problem (disregarding its frequency), evaluated on a visual analog scale (VAS) from 0 to 10.
- An Index Score was generated at each time point as previously described (26), by multiplying the frequency and intensity scores, and the change in this over time was the primary efficacy outcome for the study.

At D28 (the last day of the study), additional questions were asked to owner concerning their satisfaction, the overall efficacy and the efficacy of each different problem behavior declared at D0 by the owner. This was evaluated on a visual analog scale (VAS) from 0 to 10.

Cats were removed from analysis of the collar efficacy if:

- Collars were permanently removed (by the cat or the caregiver) before day 28 (e.g., collar broken, adverse event, decision to stop the study…).
- Collars were temporarily removed on more than two occasion and for more than 6 h each time.
- Caregivers failed to complete the online questionnaires.
- Caregivers were present in the home for < 5 days and/or < 5 evenings during the 7 days preceding each questionnaire.
- Cats were in a two-cat household where the other cat was removed from the study due to any reason and presenting inter-cat difficulties.

### Adverse events and tolerance of the collars

At each time point in the study, caregivers of cats in both the PC and CC groups were asked (see Appendix 1) to record:

- If the collar had been removed (temporarily or permanently) and if so, why.
- If the cat was not tolerating the collar well, and if not why.
- If they had encountered any unexpected event or illness with the cat, or if the cat had received any treatment, and to provide details.

Where any potential adverse reactions were identified, caregivers were contacted to obtain further details and adverse reactions were documented and coded according to the European Medicines Agency reporting criteria for suspected adverse reactions.

### Statistical analysis

Efficacy analyses were performed on the population of cats without major deviations (analyzed population in Figure 1). Safety criteria such as adverse events and tolerance issue of the collars were reported on the safety population (i.e., on cats that received a collar from the study).

Primary outcome in this study was the index score change from baseline (day 0) of each problem behavior. To compare PC and CC groups, a generalized linear mixed model (GLMM) was used on the primary outcome for the four problem behaviors. Baseline score, age in classes [≤ 2,[2;8] or > 8 years], treatment (PC vs. CC), day, day and treatment interaction were incorporated as fixed effects in the model, as well as number of cats in the households (1, 2) for scratching and excessive fears behaviors. The fact that cats had outdoor access (Yes/No) was tested for each model but finally not kept in the best model chosen according to the Akaike information criterion (AIC). Cat ID and household ID were defined as nested random effect. No adjustments for multiple comparisons were made.

Additionally, other secondary outcomes such as rate of cats that stopped the behavior at D28 and owner's opinion have been compared using Chi squared tests (χ^2^) and Wilcoxon signed-rank tests (WS) respectively on an exploratory manner with alpha level = 5%.

All statistical analyses were conducted with the use of SAS v9.4 (SAS Institute).

## Results

**Figure 2:**
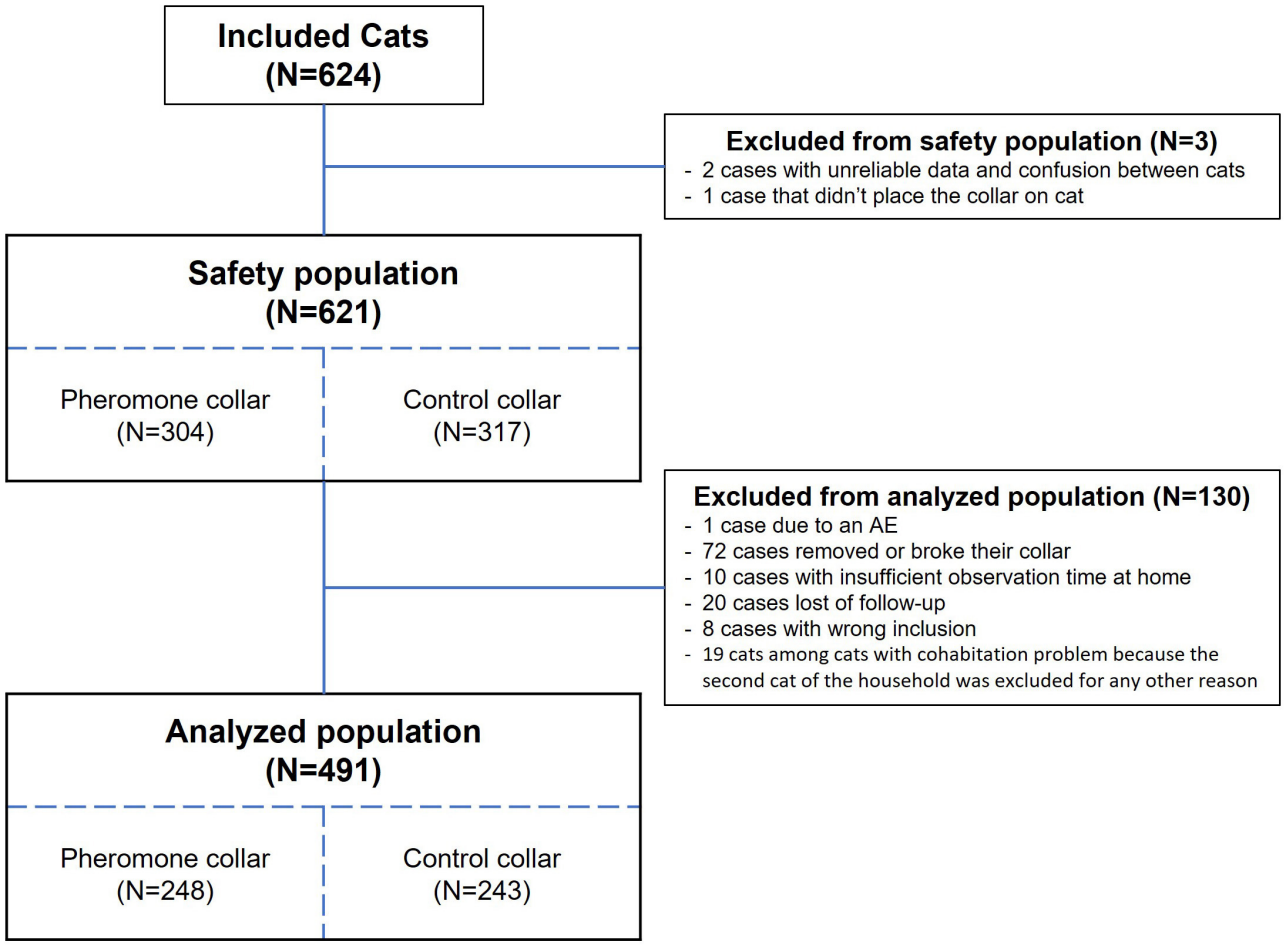
The study populations and reasons for exclusion.

In our study, a total of 624 cats from 459 households were recruited. Among this population, 3 cats were excluded from the safety analysis, and an additional 130 cats were excluded from the final efficacy analysis (see Figure 2). The analyzed population is detailed in Supplementary Table 1, and the distribution of cats between the treatment groups was similar. Since the 459 households were randomized for group allocation, there is no need for statistical testing at baseline to compare the groups (27, 28). In this study all caregivers declared that their cats tolerated collars well and around 40 % of the population wore a collar before stating this study.

**Figure 3:**
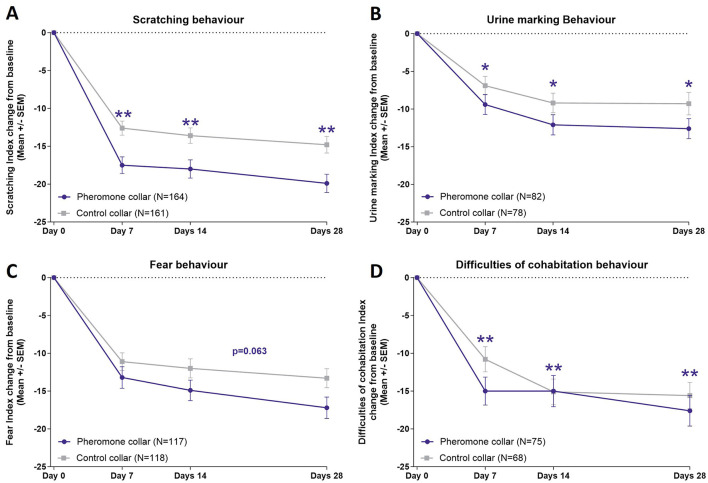
Changes in the Index Scores of problem behaviors over time. **(A)** Scratching. **(B)** Urine marking. **(C)** Fear. **(D)** Difficulties of cohabitation. Graphs show mean (and SEM) of the index change from baseline. Treatment comparison *p*-value: *GLMM *p*-value < 0.05; **GLMM *p*-value < 0.01.

Efficacy was assessed using the CABIAS^TM^, with Index values presented in Supplementary Table 2. In Figure 3, the change in Index Scores from baseline can be seen for each of the problem behaviors assessed. Statistical differences between the PC and the CC were observed for undesirable scratching behavior (GLMM: *p* < 0.01), urine marking behavior (GLMM: *p* < 0.05), and difficulties in inter-cat cohabitation (GLMM: *p* < 0.01). Although the difference for excessive fear was almost significant (GLMM: *p* = 0.063), it did not reach statistical significance. These results show a greater reduction in the Index Score (CABIAS^TM^) for the PC over the CC for the four problem behaviors and for three of them the reduction is statistically demonstrated.

**Figure 4:**
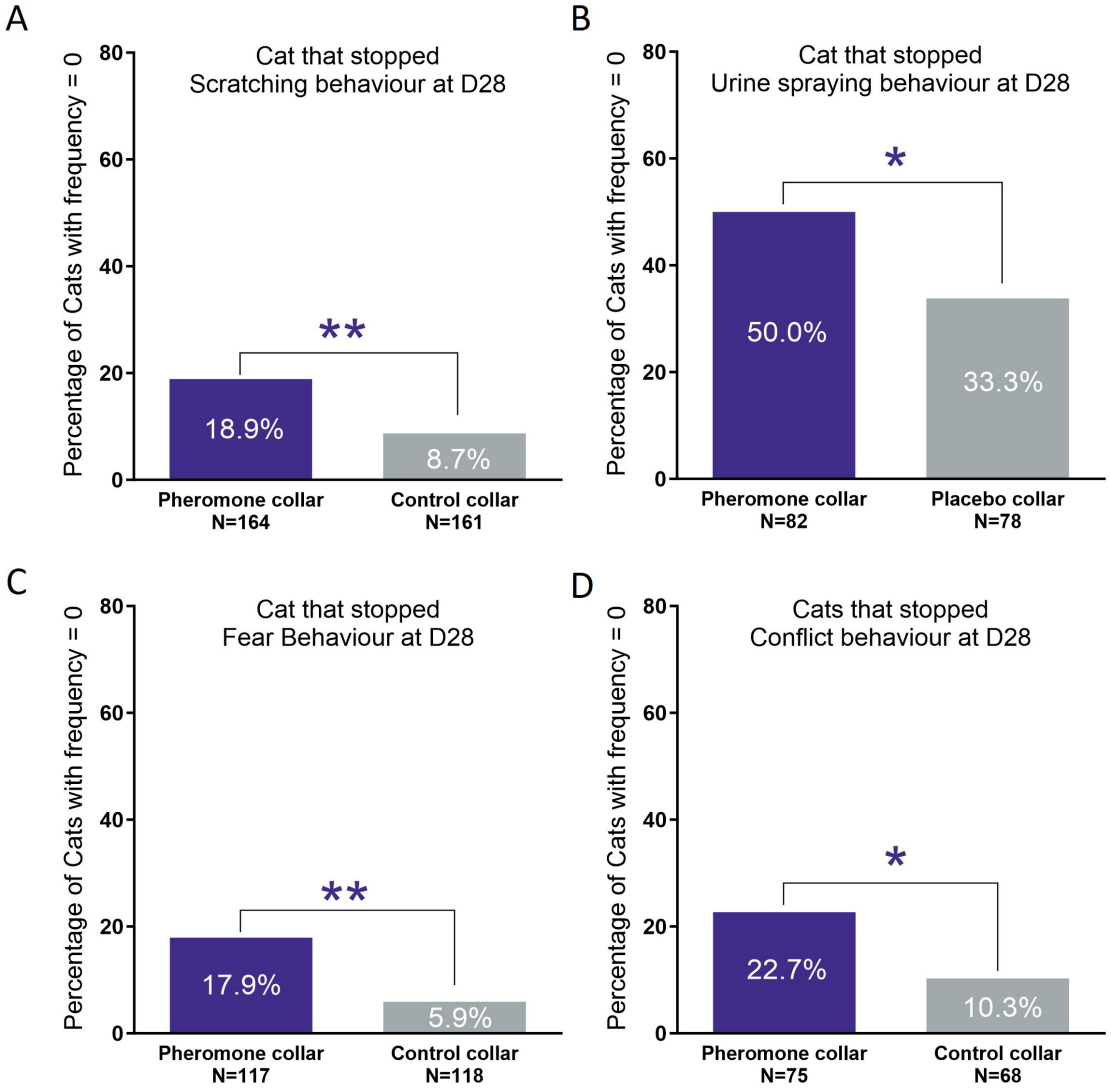
Percentage of cats that had stopped the problem behavior at the end of the study. **(A)** Scratching. **(B)** Urine marking. **(C)** Fear. **(D)** Difficulties of cohabitation. Treatment comparison *p*-value: *χ^2^-test *p*-value < 0.05; **χ^2^-test *p*-value < 0.01.

Another approach to evaluating efficacy is to compare the percentage of cats that stopped expressing these behaviors. Figure 4 illustrates that significantly more cats using the PC stopped problem behaviors after 28 days compared to the CC. In general, there were twice as many cats stopping undesirable behaviors in the PC group compared to the CC group. This provides strong evidence for the efficacy of FELIWAY Optimum pheromone collar in managing problem behavior in cats.

Finally, the owner's opinion concerning the overall efficacy and the efficacy for each problem behavior was captured using visual analog scales and compared between groups using Wilcoxon signed-rank test (see Supplementary Table 4). Significant differences were obtained for the satisfaction and the overall efficacy as well as for scratching, fear and for urine marking behavior.

The collar's safety and tolerance were evaluated on 621 cats (refer to Figure 2). The study found that 24.7% of cats with the PC and 29.7% with the CC reported at least one adverse event (see Table 1). Regardless of group, a higher percentage of adverse events was observed for cats that were not wearing a collar at baseline. In both groups, ~7–8% of cats reported an adverse event while they were wearing a collar (see Table 1).

**Table 1:** Summary of some safety parameters.

	**Pheromone collar (*N* = 304) *N* (%)**	**Control collar (*N* = 317) *N* (%)**
Number of cats with at least one Adverse event (AE)	75 (24.7%)	94 (29.7%)
Number of cats not tolerating the collar^*^	43 (14.1%)	59 (18.6%)
Number of cats with an AE followed by collar removal	29 (9.5%)	14 (4.4%)
Number of cats wearing a collar before baseline with adverse event	21 (6.9%)	25 (7.9%)
Number of cats not wearing a collar before baseline with adverse event	54 (17.8%)	69 (21.8%)
Number of cats wearing a collar at baseline	128 (42.1%)	119 (37.5%)

^*^Some caregivers reported minor issues like scratching and alopecia but felt their cats tolerated the collars. Other adverse events like vomiting and behavioral problems were not always linked to collar tolerance, explaining the difference of the percentage of cats not tolerating the collar and the percentage of adverse event.

**Figure 5:**
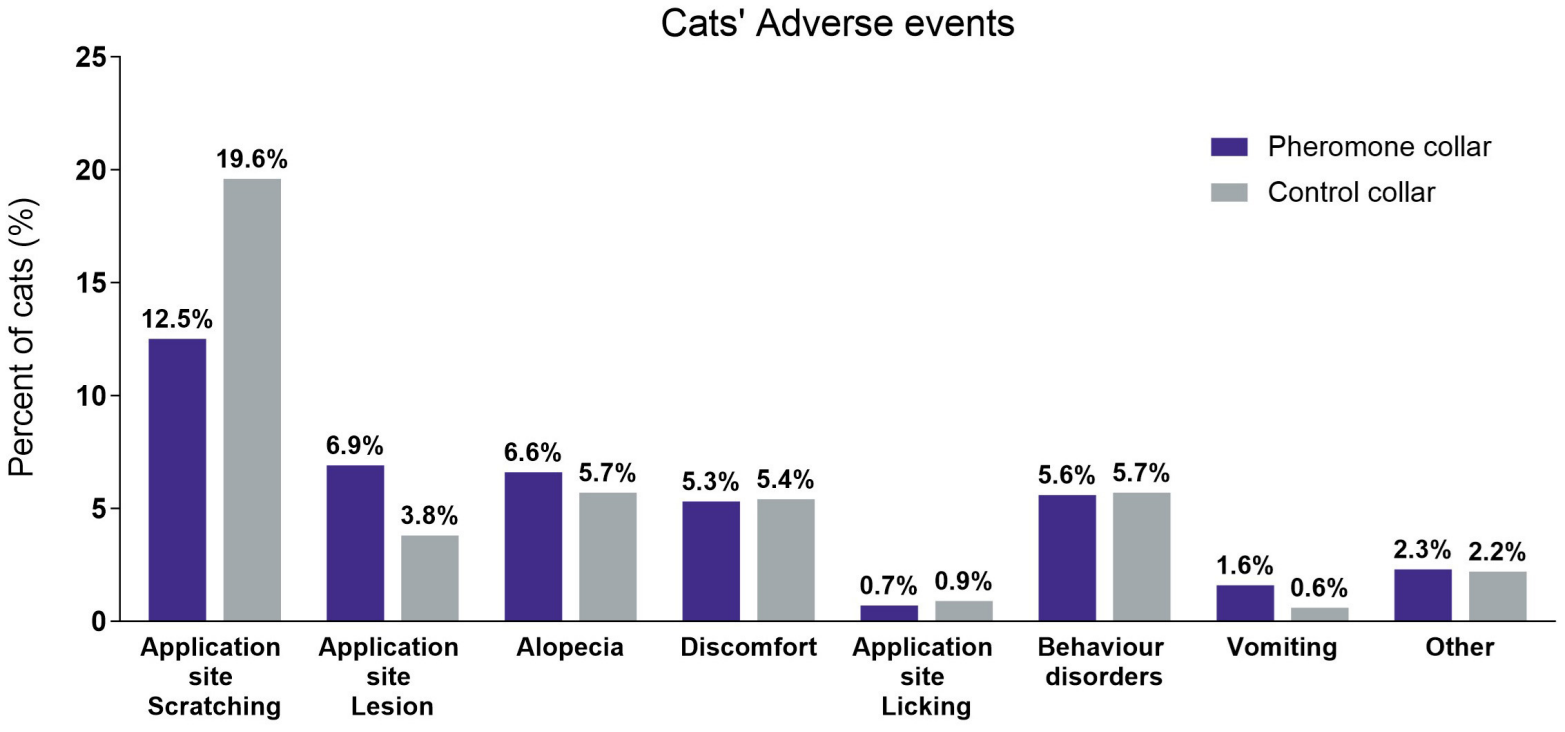
Safety. Percentage of adverse event reported on the total population. Behavior disorder includes a large variety of behavior changes, such as a cat's change in routine. Pheromone collar *N* = 304 and control collar *N* = 317.

The occurrence of unexpected events related to cat collars is diverse, but a significant proportion of these events are expected to be around the neck area where the collar comes into contact with the skin. The most commonly reported signs of general collar intolerance in this study included scratching, followed by alopecia (hair loss), discomfort, and issues at the application site. Notably, the CC group exhibited a higher proportion of scratching (19.6%) compared to the PC group (12.5%) (Figure 5). Additionally, a slightly higher percentage of cats with skin lesions were observed in the PC group (6.9%) compared to the CC group (3.8%).

## Discussion

In the current study, a randomized placebo-controlled design was used to assess the efficacy of FELIWAY^®^ *Optimum* (Ceva Santé Animale), in addition a novel delivery method was employed with the pheromone complex being impregnated in a polymer collar designed to release the product over 28 days. The results showed that the problem behaviors improved over the duration of the study, and compared with the control group, a greater improvement was seen in all four problem behaviors in cats wearing the pheromone collar, which was statistically significant for all except problem fear. As identified in previous studies, the placebo effect when investigating problem behaviors can be substantial, and so this study provides important additional evidence of the efficacy of the novel feline pheromone complex as a tool to help manage a variety of problem behaviors.

Quantitative or semi-quantitative assessment of problem behaviors in cats in a normal household setting is inherently problematic. Some behaviors (such as fear, or inter-cat aggression) can rely on direct observation of events by caregivers, but with others (such as inappropriate urination or scratching) there may be indirect evidence as well. In consequence, to be eligible for results to be included in this study, we therefore sought to ensure caregivers were present in the home for at least 5 days and/or evenings during the 7 days prior to each of the assessment times. Additionally, as before, we asked caregivers to estimate both the frequency and their perception of the intensity or severity of the problem (disregarding its frequency) during the previous 7 days at each time point, and we used the Index Score (frequency x intensity) as the main efficacy outcome measure (18, 26). Although different caregivers are likely to assess the severity of a problem behavior differently, the fact that this was a placebo-controlled longitudinal study, looking at changes within each cat over time, helps overcome this issue. Additionally, at each time point caregivers had to assess the problem behavior “blinded” to their previous scores to avoid introducing bias. Behavior assessments in veterinary science are usually reported by the owner. Although we recognize that technological advancements, such as video recording, can provide direct observation, this method would have required video collection from many rooms in a participant's house, which was deemed nearly impossible given the number of cats included in this study. Implementing such technology in participants' homes could also pose ethical and privacy concerns. To address the potential lack of accuracy due to owner reporting, we included a large number of cats in the study. Additionally, we used previously validated scales that allow for the observation and recording of behavioral changes.

The results of this study provide strong evidence for the efficacy of the pheromone complex, especially as no other changes were introduced to help manage the problem behaviors as would normally happen in clinical practice. This study also demonstrated the viability of delivering feline pheromones through a therapeutic collar, and the importance of using a control collar group was not only to assess the efficacy of the pheromone complex, but also to look at the ease of use and tolerance of the collars.

Of the 621 cats of the safety population, the collar was lost or definitively removed in 12.1% before the end of the study. That proportion is similar to a study evaluating three different types of regular collars in cats (30), but in that longer-term study the proportion of collars lost or removed continued to increase to a mean of >27% at 6 months. It is therefore clear that removal of collars (by the cat or the caregiver, for various reasons) may be an appreciable problem (30–32), and this could impact the use of any form of collar. Interestingly, we found no cases where a cat had got its forelimb trapped in the collar, a well-recognized but relatively uncommon problem with collars (25, 30, 31, 33). This might in part be due to the short duration of this trial, but perhaps also because caregivers were asked to check and adjust (if needed) the fit of the collar each time they completed a questionnaire. It worth also notice that 6.9% of the cats had their collar removed following an adverse event and a higher percentage of adverse events was observed for cats that were not wearing a collar at baseline.

Unfortunately, the incorporation of the pheromone complex altered the physical properties of the polymer collar, so a “like-for-like” placebo could not be used. Instead, we chose a commercially available silicone collar that had similar flexibility to the pheromone-impregnated collar, that might have influence the ease of use and solidity between the two collars. Lord et al. (30) reported that structural differences between three different regular collars affected both the ease of use and frequency of loss of collars in their study.

In this study, besides the fact that cats were selected in part due to their caregiver's assessment that they tolerated collars well, caregiver more than 26% reported skin problems (included scratching, alopecia and lesion where the collar was). These values are similar to a previous study where 27% of caregivers reported issues with collar (33).

The range of adverse reactions reported in association with the use of both collars was very similar to those reported previously (25, 30, 31, 33). There was no difference in the overall prevalence of adverse reactions between the pheromone and control collars, but interestingly adverse reactions were the cause of a higher number of the pheromone collars being removed by caregivers, and a higher proportion of pheromone collars were associated with development of lesions while control collar provoked more scratching reaction. This may reflect differences in the physicochemical properties of the two collars, for example, the pheromone collar had notches on the inner surface which might rub the skin, whereas the control collar appeared to provoke more scratching reactions. Skin lesions, irritation and hair loss are well-recognized problems associated with a wide variety of collars used in cats (32) which was also seen with the control collar used in this study. Although these local effects are sometimes assumed to reflect hypersensitivity reactions, they may equally reflect mechanical damage and self-inflicted injury due to collar irritation (25).

## Conclusion

This study is the first randomized placebo-controlled study to report on the efficacy of the novel pheromone complex FELIWAY^®^ *Optimum* (Ceva Santé Animale), and the first to report on the prevalence of adverse events associated with the use of a pheromone collar in a large (>500) population of cats. We were able to demonstrate the superior efficacy of the pheromone-impregnated collars in helping to improve three of the four problem behaviors evaluated (problem urination, scratching and inter-cat conflict) with a non-significant trend for improvement in problem fear as well. This provides good evidence to support the use of FELIWAY^®^ *Optimum* (Ceva Santé Animale), especially if incorporated as part of a multimodal approach to management of problem behaviors (15, 16, 34).

Although physical and structural differences between the pheromone and control collars appeared to result in some differences in problems reported, there was no evidence that the pheromone-impregnated collar resulted in any overall higher level of adverse events. Nevertheless, the proportion of cats presenting adverse event at the end of the study (for a variety of reasons) suggests this might not be the most effective way of delivering the feline pheromone complex and provide evidence again concerning the discomfort and skin irritation for cats wearing a collar.

## Data Availability

The original contributions presented in the study are included in the article/Supplementary material, further inquiries can be directed to the corresponding author.
