# Supplementary material for: Efficacy of a pheromone-impregnated collar in controlling feline problem behaviors, and an assessment of adverse events associated with collar use
Source: Front Vet Sci. 2024 Dec 19;11:1468634. doi: 10.3389/fvets.2024.1468634 (PMC11694449; doi:10.3389/fvets.2024.1468634)
Supplement: Supplementary file 1 [file Data_Sheet_1.pdf]

Supplementary Material

1 Supplementary Table

Supplementary Table 1. Description of the analyzed population. * For five households, only one cat of the two present in the household was kept in the analysed population. ** Percentages based on households with at least one cat in the analyzed population.

Category	Item	Pheromone collar (n=248)	Placebo collar (n=243)
**Cats in household ***	One	131 (52.8%)	125 (51.4%)
	Two	117 (47.2%)	118 (48.6%)
**Sex**	Male	121 (48.8%)	100 (41.2%)
	Female	127 (51.2%)	143 (58.8%)
**Age (years)**	Median	5.0	5.0
	Range	0.6-17	0.8-17.0
**Weight (kg)**	Median	4.2	4.0
	Range	2.5-11.0	2.5-12.0
**Outdoor access ****	None	38 (19.9%)	48 (25.9%)
	Restricted	105 (55.0%)	96 (24.9%)
	Free access	48 (25.1%)	41 (22.2%)
**Number of problems**	One	106 (42.7%)	105 (43.2%)
	Two	99 (39.9%)	101 (41.6%)
	Three	38 (15.3%)	30 (12.3%)
	Four	5 (2.0%)	7 (2.9%)
**Problem behaviours**	Urination	82	78
	Scratching	164	161
	Fear	117	118
	Conflict	75	68

Supplementary Table 2. Index scores for the four problem behaviours over time

	Index score	Pheromone Collar group		Control Collar group	
		Median	IQR	Median	IQR
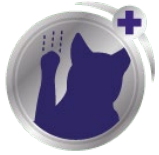	**Problem scratching**	**n=164**		**n=161**	
	Day 0	33.0	20.1 – 44.7	30.0	18.3 – 40.5
	Day 7	12.2	4.4 – 22.2	14.5	6.8 – 24.5
	Day 14	10.9	2.3 – 23.4	13.2	4.4 – 26.8
	Day 28	8.6	1.1 – 19.1	11.7	2.6 – 23.0
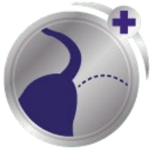	**Problem urination**	**n=82**		**n=78**	
	Day 0	12.6	6.7 – 21.0	10.4	5.1 – 20.1
	Day 7	4.1	0.0 – 10.8	4.6	2.0 – 11.8
	Day 14	1.7	0.0 – 4.0	3.2	0.0 – 9.8
	Day 28	0.5	0.0 – 4.7	2.3	0.0 – 6.5
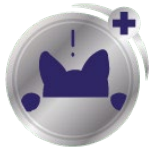	**Problem fear**	**n=117**		**n=118**	
	Day 0	30.0	13.5 – 41.4	30.1	15.3 – 41.4
	Day 7	10.2	4.4 – 22.8	13.8	7.2 – 28.0
	Day 14	8.4	3.0 – 20.4	13.6	4.4 – 24.9
	Day 28	5.7	1.6 – 18.6	13.6	3.9 – 24.8
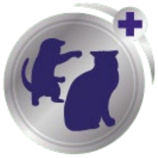	**Problem conflict**	**n=75**		**n=68**	
	Day 0	25.0	13.5 – 41.5	34.2	26.2 – 41.5
	Day 7	9.2	2.2 – 19.6	21.0	10.1 – 33.8
	Day 14	8.2	2.0 – 22.0	19.5	6.0 – 27.6
	Day 28	4.7	0.7 – 16.2	14.7	5.6 – 30.2

Supplementary Table 3. Index scores expressed as absolute change from baseline for the four problem behaviours over time

	Absolute change from Baseline	Pheromone Collar group	Control Collar group
		Mean ± SD	Mean ± SD
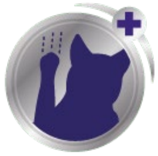	**Problem scratching**	**n=164**	**n=161**
	Day 0		
	Day 7	-17.5 ± 14.2	-12.6 ± 11.9
	Day 14	-18.0 ± 15.4	-13.6 ± 13.0
	Day 28	-19.9 ± 15.5	-14.8 ± 13.8
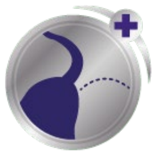	**Problem urination**	**n=82**	**n=78**
	Day 0		
	Day 7	-9.4 ± 12.1	-6.9 ± 10.8
	Day 14	-12.1 ± 12.2	-9.2 ± 11.5
	Day 28	-12.6 ± 12.1	-9.3 ± 13.2
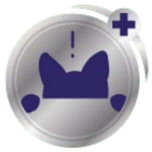	**Problem fear**	**n=117**	**n=118**
	Day 0		
	Day 7	-13.2 ± 15.4	-11.1 ± 12.7
	Day 14	-14.9 ± 14.6	-12.0 ± 13.8
	Day 28	-17.2 ± 15.3	-13.3 ± 13.8
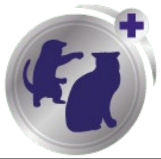	**Problem conflict**	**n=75**	**n=68**
	Day 0		
	Day 7	-15.0 ± 16.0	-10.8 ± 13.7
	Day 14	-15.0 ± 17.8	-15.1 ± 13.9
	Day 28	-17.6 ±17.5	-15.6 ± 14.5

Supplementary Table 4. Caregiver judgment of overall satisfaction. Caregiver evaluation of efficacy globally and for each problem behaviour per household

			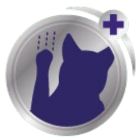	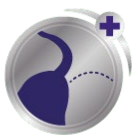	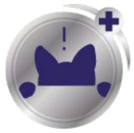	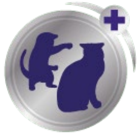
Mean ± SD	**Product satisfaction**	**Product Efficacy**	**Scratching Efficacy**	**Urine Marking Efficacy**	**Fears Efficacy**	**Tension and conflicts Efficacy**
Pheromone Collar	7.1 ± 2.6	7.0 ± 2.5	6.7 ± 2.8	7.6 ± 2.4	6.6 ± 2.8	6.4 ± 2.8
Control Collar	6.6 ± 2.7	6.2 ± 2.9	5.9 ± 2.9	6.5 ± 2.9	5.6 ± 2.9	5.8 ± 3.1
WS p-value	p<0.05	p<0.01	p<0.05	p<0.05	p<0.05	NS

Recruitment Questionnaire

1 Do you agree to adhere to this confidentiality agreement?
1. Yes
2. No STOP if 2 is selected
3.

2 Have you participated in a study in the last 3 months?

1. Yes
2. No

2 bis. (If code 2 in Q2) For which type(s) of products have you conducted a study in the last 3 months? (Multiple responses possible, randomly ordered)
1. Pet products / pet food
2. Food products
3. Beverages
4. Cleaning products
5. None of these answers STOP if 1 is selected

3 Do any of the following situations apply to you? (Multiple responses possible)
1. You are part of a panel dedicated to a specific brand or manufacturer
2. You are an expert in sensory analysis
3. None of these answers STOP if 1 or 2 is selected

4 Do you or any of your relatives work in any of the following fields? (Multiple responses possible)
1. Agro-food
2. Banking / Finance / Insurance
3. Pharmaceutical industry STOP
4. Cosmetic industry
5. Veterinary practice / clinic STOP
6. Pet store STOP
7. Market research institute STOP
8. Advertising / marketing / communication agency STOP
9. Journalism / media STOP
10. Automotive industry
11. Manufacturing or distribution of pet food or products STOP
12. None of these fields

5 How old are you? STOP if under 18 years old

6 Are you?
1. A woman
2. A man

7 What is your postal code? /______/

7 Bis. Do you live?
1. In a city/urban area
2. In a village
3. In the countryside

7 Ter. Do you live?
1. In an apartment with a garden / terrace
2. In an apartment without outdoor space
3. In a house with a garden
4. In a house without outdoor space

8 Which of the following best represents your family situation? (Only one answer possible)
1. Single without children at home
2. Single with children at home
3. Married or living with a partner without children at home
4. Married or living with a partner with children at home

9 What is your professional category? (Only one answer possible)
1. Farmer
2. Artisan, Shopkeeper, Business owner
3. Executive and higher intellectual profession
4. Intermediate profession
5. Employee
6. Worker
7. Retired
8. Other inactive

10 Generally, during the week, excluding evenings, weekends, and holidays, how much time do you spend at home?
1. I spend almost all my days at home (teleworking, homemaker, etc.)
2. I spend 1 day outside and 4 days at home
3. I spend 2 or 3 days at home and 2 or 3 days outside
4. I spend 1 day at home and 4 days outside
5. I spend all my days outside

11 Do you have any pets at home?
1. No
2. Yes STOP if 1 is selected

12 What pet(s) do you have at home? (Multiple responses possible)
1. Dog(s)
2. Cat(s)
3. Others (specify) STOP if 2 is not selected

13 How many cats do you have at home? (Only one answer possible)

1. 1
2. 2
3. 3 or more STOP if code 3 is selected

14 Are you the one in the household responsible for the cat's health care (e.g., vet visits, medication administration, etc.)?

1. Yes
2. No STOP if 2 is selected

15 Are your 2 cats' vaccination records up to date?

1. Yes
2. No STOP if 2 is selected

16 What is the age of your cat(s)? (If more than 1 cat: Multiple responses possible)

1. Less than 6 months
2. From 6 months to 12 years
3. More than 12 years STOP if 1 or 3 is selected

17 Bis. How much does your cat(s) weigh? (If more than 1 cat: Multiple responses possible)

1. Less than 2.5 kg
2. From 2.5 kg to 5 kg
3. More than 5 kg STOP if 1 is selected

18 Does your cat(s) currently have a disease diagnosed by a veterinarian?

1. Yes
2. No STOP if 1 is selected

19 Bis. Do you or anyone else in your household suffer from an allergy?

1. Yes
2. No STOP if 1 is selected

20 How many cat litters do you have at home? (Only one answer)

1. None
2. 1
3. 2
4. 3
5. 4
6. 5 or more STOP if code 1 OR (if code 2 in Q14 + code 2 in Q19)

21 How many cat trees/scratch posts do you have at home? (Only one answer)

1. None
2. 1
3. 2
4. 3
5. 4
6. 5 or more STOP if code 1

22 How many food bowls do you have at home for your cat(s)? (Only one answer)
1. None
2. 1
3. 2
4. 3
5. 4
6. 5 or more STOP if code 1 OR (if code 2 in Q14 + code 2 in Q21)

23 Has your cat (one of your cats) been hospitalized in the last 15 days (even for just one day)?
1. Yes
2. No STOP if 1 is selected

24 Which of the following best describes your cat(s) lifestyle? (Only one answer)
1. My cat(s) has (have) controlled access to the outside
2. My cat(s) has (have) unlimited
3. My cat(s) does/do not have access to the outside

24. Which of the following behavioral problems apply to at least one of your cats? (Select all that apply)

1. Urine marking: urinating inside, outside the litter box while standing (on a vertical surface)
2. Scratching inside on vertical surfaces other than the cat tree, e.g., the sofa, furniture, curtains, door frames…
3. Excessive fear, including excessive hiding (e.g., following situations like a visitor in your home, children playing near the cat, unusual noise, a noisy moment, or an unusual situation)
4. (If code 2 in Q14) Difficulty cohabiting with other cats in your household indoors (fights, conflicts, attacks, intimidation, chasing, blocking passage)
5. Other behavioral problem (specify)
6. My cat(s) does/do not have any particular behavioral problems STOP if codes 1, 2, 3, or 4 are not mentioned

24. Bis. Has at least one of these problems appeared more than a month ago?

1. Yes
2. No STOP if code 2

If multiple answers in Q24

25. Among the behavioral problems that appeared more than a month ago, which one concerns you the most? (Select one answer)

1. Urine marking: urinating inside, outside the litter box while standing (on a vertical surface)
2. Scratching inside on vertical surfaces other than the cat tree, e.g., the sofa, furniture, curtains, door frames…
3. Excessive fear, including excessive hiding (e.g., following situations like a visitor in your home, children playing near the cat, unusual noise, a noisy moment, or an unusual situation)
4. (If 2 cats in the household) Difficulty cohabiting with other cats in your household indoors (fights, conflicts, attacks, intimidation, chasing, blocking passage)

25. Bis. How long has this problem of urination/scratching/excessive fear/difficulty cohabiting been present? Dropdown menu month/year

26. To what extent is this behavioral problem disturbing for you and your household? (from "not at all disturbing" to "extremely disturbing")

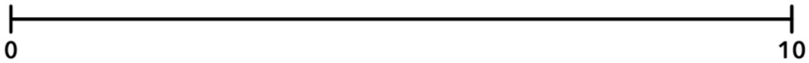

27 To what extent are you motivated to tackle this behavioral problem? (from "I am not motivated, I am used to it" to "I am extremely motivated")

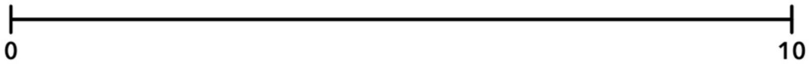

28 Are you currently using OR have you used any of the following products in the last 6 months to help combat your cat(s)' behavioral problems?

1. Anti-stress drops/tablets
2. FELIWAY CLASSIC diffuser
3. FELIWAY CLASSIC spray
4. FELIWAY FRIENDS diffuser
5. FELIWAY OPTIMUM diffuser
6. Beaphar Cat Comfort
7. Catizen
8. Zensylk
9. Zenifel
10. Francodex Antistress
11. Nutraceutical or medical calming products
12. Other calming products, please specify
13. Don't know
14. None of these products STOP if 14 is not mentioned

If 1 cat IN THE HOUSEHOLD (code 1 in Q14)

29 What is your cat’s name? / __________________/

30 Is your cat currently wearing a collar?

1. Yes
2. No

31 If yes in Q30, what type of collars is your cat currently wearing? (Multiple answers possible)

1. Anti-parasite, anti-flea collar
2. GPS collar
3. Collar with tag/identification
4. Fancy collar
5. Other (specify)

33 If no in Q30, has your cat ever worn a collar?

1. Yes
2. No If no, STOP

34 If yes in Q31, what type of collar has your cat worn? (Multiple answers possible)

1. Anti-parasite, anti-flea collar
2. GPS collar
3. Collar with tag/identification
4. Fancy collar
5. Other (specify)

35 If yes in Q32, does your cat tolerate wearing a collar well?

1. Yes
2. No If no, STOP

For this test, you will be asked to test a collar for cats. This pheromone-based collar aims to help your cat naturally manage and reduce behaviors such as scratching, urine marking, excessive fears, and cohabitation issues between cats. It has already been tested and poses no danger to your pet. Additionally, it has a safety feature in case your cat gets stuck. If your cat is already wearing a collar, it will need to wear the test collar in addition to its usual collar. Note that in this study, by random draw, you may receive a product without an active ingredient for comparison purposes.

36 Do you agree to test this collar on your cat to help combat its behavioral problems for a duration of 4 weeks?

1. Yes
2. No STOP if 2 is mentioned

If 2 cats in the household (code 2 in Q14)

You have declared having 2 cats at home. We would like to know more specifically about the behavior of each of your cats.

Questions asked per cat

38. What is the name of your (first/second) cat? / ___________________/

39. Among the behavioral problems below, which one(s) specifically concern [name given in Q38]? (Multiple answers possible)

1. Urine marking: urinating inside, outside the litter box while standing (on a vertical surface)
2. Scratching inside on vertical surfaces other than the cat tree, e.g., the sofa, furniture, curtains, door frames…
3. Excessive fear, including excessive hiding (e.g., following situations like a visitor in your home, children playing near the cat, unusual noise, a noisy moment, or an unusual situation)
4. Difficulty cohabiting with other cats in your household indoors (fights, conflicts, attacks, intimidation, chasing, blocking passage)
5. Other behavioral problem (specify)
6. My cat(s) does/do not have any particular behavioral problems

If codes 1, 2, 3, or 4 are mentioned and if multiple answers in Q39

40. Among the behavioral problems of [name given in Q38] that you just indicated, which one bothers you the most? (Select one answer)

1. Urine marking: urinating inside, outside the litter box while standing (on a vertical surface)
2. Scratching inside on vertical surfaces other than the cat tree, e.g., the sofa, furniture, curtains, door frames…
3. Excessive fear, including excessive hiding (e.g., following situations like a visitor in your home, children playing near the cat, unusual noise, a noisy moment, or an unusual situation)
4. Difficulty cohabiting with other cats in your household indoors (fights, conflicts, attacks, intimidation, chasing, blocking passage)

41. Does [name given in Q38] currently wear a collar?
1. Yes
2. No

42. If yes in Q41, what type of collars does your cat currently wear? (Multiple answers possible)

1. Anti-parasite, anti-flea collar
2. GPS collar
3. Collar with tag/identification
4. Decorative collar
5. Other (specify)

43. If no in Q41, has [name given in Q38] ever worn a collar?
1. Yes

2. No If no, STOP

44. If yes in Q41, what type of collar has your cat worn before? (Multiple answers possible)
    1. Anti-parasite, anti-flea collar
    2. GPS collar
    3. Collar with tag/identification
    4. Decorative collar
    5. Other (specify)

45. If yes in Q41, does [name given in Q38] tolerate wearing a collar well?
    1. Yes
    2. No If no, STOP

For this test, you will be asked to test a collar for cats. This pheromone-based collar aims to help your cat naturally manage and reduce behaviors such as scratching, urine marking, excessive fears, and cohabitation issues between cats. It has already been tested and poses no danger to your pet. Additionally, it has a safety feature in case your cat gets stuck. If your cat already wears a collar, it will need to wear the test collar in addition to its usual collar.

Please note that in this study, by random draw, you may receive a product without an active ingredient to serve as a comparison.

[If codes 1 to 4 are not mentioned in Q39] For this study, both cats in the household must test a collar even if one of them does not have any particular behavioral problems.

46. Do you agree to test this collar on your cat [name given in Q38] for 28 days?
    1. Yes
    2. No If no, STOP

2 Questionnaire: Day 1 – summary of questions asked

2.1 Owner presence in home

1. In the last 7 days how many days were you present in your home? (0-7)

2. In the last 7 days how many evenings were you present in your home? (0-7)

2.2 Problem behaviour (questions asked for each cat and each problem)

3. In the last 7 days, how many times have you noticed your cat has displayed the problem behaviour (urine marking / scratching / excessive fear / difficulties of cohabitation)?
   7. Every day, more than twice daily
   6. Every day, once or twice daily
   5. Almost every day
   4. Every other day
   3. Twice a week
   2. Once a week
   1. Never

4. Disregarding the frequency, how would you rate the average intensity of the problem behaviour (urine marking / scratching / excessive fear / difficulties of cohabitation)? Place the cursor on this scale:

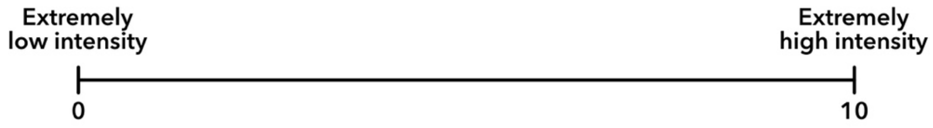

2.3 Use of the collar

5. Were you able to put the collar around your cat's neck? ………………… ☐ Yes ☐ No

6. If you could not place the collar, please explain why: ________________________________

7. If your cat did not tolerate the collar well, please explain why: _________________________

2.4 Relationship with the cat

8. How would you rate the current happiness of your cat? Place the cursor on this scale:

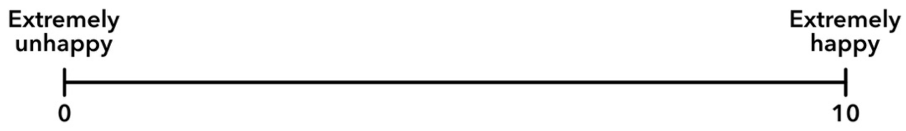

9. How would you describe your current relationship with your cat? Place the cursor on this scale:

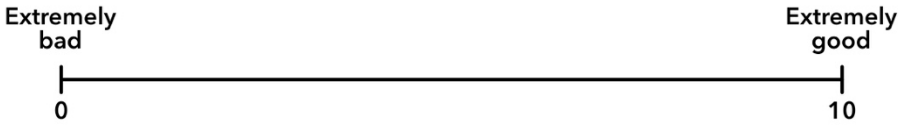

10. Over the last 7 days, approximately how many times has your cat actively sought your affection?
    1. Less than 3 times
    2. Every other day
    3. Once daily
    4. Twice daily
    5. 3-4 times daily
    6. 4-6 times daily
    7. More than 6 times daily

11. How long would you say your cat typically tolerates being stroked
    1. A short time
    2. A fairly short time
    3. Neither a short or long time
    4. Quite a long time
    5. A very long time

Questionnaire: Days 0, 7, 14 and 21 – summary of questions asked

2.5 Problem behaviour (questions asked for each cat and each problem)

In the last 7 days, how many times have you noticed your cat has displayed the problem behaviour (urine marking / scratching / excessive fear / difficulties of cohabitation)?

7. Every day, more than twice daily
6. Every day, once or twice daily
5. Almost every day
4. Every other day
3. Twice a week
2. Once a week
1. Never

Disregarding the frequency, how would you rate the average intensity of the problem behaviour (urine marking / scratching / excessive fear / difficulties of cohabitation) in the last 7 days? Place the cursor on this scale:

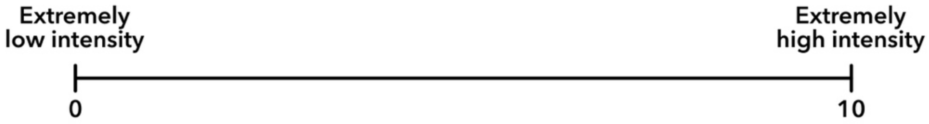

2.6 Relationship with the cat

How would you rate the current happiness of your cat? Place the cursor on this scale:

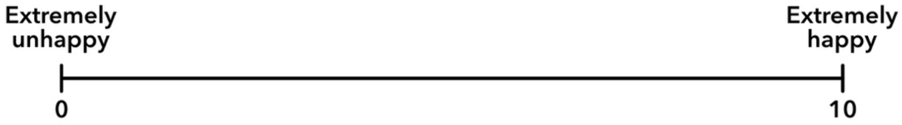

How would you describe your current relationship with your cat? Place the cursor on this scale:

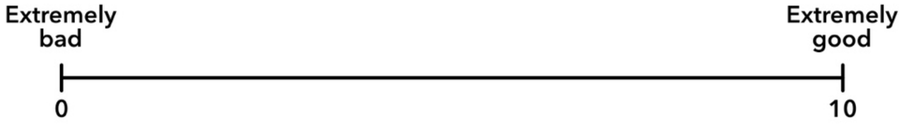

Over the last 7 days, approximately how many times has your cat actively sought your affection?

1. Less than 3 times
2. Every other day
3. Once daily
4. Twice daily
5. 3-4 times daily
6. 4-6 times daily
7. More than 6 times daily

How long would you say your cat typically tolerates being stroked

1. A short time

2. A fairly short time
3. Neither a short or long time
4. Quite a long time
5. A very long time

Questionnaire: Days 7, 14 and 21 – summary of questions asked

2.7 Other problems

Since the last questionnaire, has there been any problem with your cat? …. ☐ Yes ☐ No

If your cat has had a problem, please give details (date, problem, duration): __________________

Since the last questionnaire, has your cat received any treatment? ……… ☐ Yes ☐ No

If your cat has received treatment, please specify: _______________________________________

2.8 Owner's presence in home

In the last 7 days how many days were you present in your home? (0-7)

In the last 7 days how many evenings were you present in your home? (0-7)

2.9 Use of the collar

Since the last questionnaire, have you or your cat removed the collar?

☐ Yes, temporarily ☐ Yes, permanently ☐ No

If 'Yes', please provide details (when, reasons, length of time, number of times): ______________

If your cat has not tolerated the collar since the last questionnaire, please explain why: _________
